# Editorial Notes

**Published:** 1905-12

**Authors:** 


### Editorial Notes.

**University College, Bristol. Faculty of Medicine.**

THE annual distribution ot prizes to the students of the Faculty of Medicine, on October 26th, 1905, under the chairmanship of the Right Honourable Lewis Fry, the Chairman of the Council, was this year distinguished by the presence of the Regius Professor of Medicine at Oxford, Dr. William Osler, whose assistance was much appreciated by his medical colleagues in Bristol, and whose address is one which will long be remembered. Professor Osler is well trained in the giving of such addresses, and few men have had such manifest tokens of the warm appreciation of his professional colleagues than fell to him at the great dinner given in New York just before his departure for England, when it was said justly of him that he had left a deep impress of his enthusiastic teaching in the medical circles of Montreal, Philadelphia, and Baltimore. Professor Osler's reply on that occasion is worthy to be remembered. He said: "I have three personal ideals—First, to do to-day's work well, and let to-morrow take care of itself; secondly, to act the golden rule toward my professional brothers and my patients; thirdly, to cultivate a certain measure of equanimity, to meet success with equanimity, to meet grief, pain, anguish and suffering with equanimity befitting a man. . . . I shall not worry so long as I carry the memory of the past you have given me. My mistakes have been of the head, not of the heart. I have loved no darkness, sophisticated no truth, nursed no delusion, and showed no fear."

His address to the Bristol students was as follows:—

"He had come to Bristol to talk as a student to students. He should like to express his thanks to the Faculty for the great honour they had done him in asking him to be present in a city which was associated with such men as Symonds, Prichard, Carpenter, Greig Smith, and Long Fox. To distribute the prizes at a school with which these men were connected was a distinction which he appreciated very much.

He wished to speak of the dual aspect of the student's life—first, of his professional education, of which the members of the Faculty had charge ; and, secondly, of his inner platonic education, of which the student was alone the custodian. If they wished to become successful practitioners, three things were requisite. The first was interest in their studies. They could do nothing in life unless they had an absorbing, a deep, and an abiding interest in their work. They had entered upon a profession of surpassing interest, and one of remarkable variety; indeed, it was one in which some considered there were rather too many interests. That was one of its great charms, however, its extraordinary variety in studies and research far surpassing that of any other profession. The second thing requisite for the student was industry. That was the one talent a man must have if he was to succeed in the profession. It was industry, industry, industry which alone insured success. If he had had any success in life it had been by the use of one talent—he only had one—the simple and single talent of inherited industry, without which no student could do well. The third thing essential was good health. They might smile at that statement, particularly the robust. Still, he would warn them that they could not neglect their health as medical students. Nor could they as doctors. It was a hard profession, even for the healthiest amongst them. How often they found the robust and strong in health as students failing in life because they neglected their health. Health being so essential, he would urge them thus early to take care of their health, to form good habits, to take plenty of exercise and plenty of fresh plain food. If they did this, even the delicate amongst them might find it the best thing for them. It was the second part of their education which was a much more serious and a much more important affair. It was the inner education, which went to form character, which was so much harder to acquire in this life. Yet there was no profession which required it so much as the medical profession. They might have science and all the endowments of the head, brilliant and deep; but if they had no endowments of the heart, if they had no culture, it was 'the sounding of brass and the tinkling of cymbals.' That was not the education which befitted a man for their profession. It was said that young men had too much to learn. So it might seem hard when he asked them to devote a certain part of their time to the cultivation of their characters. Many of them obtained their culture from contact with their teachers; but they, as medical students, could get it as well from good literature. It was an easy matter to devote one half-hour a day to the cultivation of their inner selves. To do this successfully they would have to start early, for they rarely saw a man attain to a high degree of culture who began after he had reached the age of 25. They should not strive to get what they could out of life, but what they could give to life. If they learned that, they would have learned the highest lesson they could learn, for they would have got a sane and sensible outlook on the world."

**Proposed University for Bristol.**

PROF. OSLER'S remarks on this subject are most helpful in the cause of University Extension. He said: "They occupied a peculiar position in Bristol, inasmuch as their Medical School was the only Medical School in Great Britain not in connection with a University. The question was whether Bristol people had risen to the importance and thought—at any rate the preparedness for a University with which this Medical School could be incorporated. There could be no question about the advantages to be derived from such a course, and he alluded to the remarkable growth of the Medical Schools in Manchester and other large centres. There were three essentials to the carrying out of such a scheme—capital, men, and buildings. He explained how a continental town with which he was acquainted, and which bore some resemblance to Bristol, save that it was not a seaport, contributed £80,000 a year for its University. That was only the interest on £2,000,000; and was it too great a thing for Bristol to raise, say, £40,000 a year? It was so much better to give their surplus money away in this manner than hoarding it, and the sum he had named did not appear too large to ask for in this wealthy community. The capital should be forthcoming which should put their University at once in a position in the very forefront of the Universities of the world. When they had secured the capital they should not encroach too much upon it for buildings. They should then look for the men, for if the men were worth much it did not matter a great deal about the buildings. There were four kinds of Universities—first, those which had buildings, but neither men nor money; second, those with men, but precious little money—these often did the very best work; third, those with money and no men; and, fourth, those like the one which they might establish in Bristol—a University with buildings, money, and men, which would be worthy of the empire."

**Faculty of Medicine.**

WE observe with great regret the resignation by Dr. E. Markham Skerritt of the posts of Dean of the Medical Faculty and Professor of Medicine at University College, Bristol. Dr. Skerritt, first as lecturer on and then professor of medicine, has been connected with the Medical School for a period of close upon thirty years. During the greater part of that time he has also acted as Dean, and since the retirement of the late Dr. J. G. Swayne, some years ago, as Chairman of the Faculty. In these capacities Dr. Skerritt worked with indefatigable energy for the good of the Medical School, which owes a great debt of gratitude to him for his untiring labours on its behalf, and also for the wisdom with which he directed its affairs, and the courtesy and tact with which he presided over the meetings of the Faculty. He retires with the sincere regret and esteem of his colleagues, and in recognition of his many and great services to the Medical School during so many years he has been appointed Emeritus Professor of Medicine in the College.

Those who know how much good work Professor Edward Fawcett has done for the School since he has occupied the Chair of Anatomy will be pleased to hear that he has accepted the office of Dean, and will feel that the interests of the Medical School are in competent hands.

Dr. Michell Clarke has been appointed Chairman of the Faculty and Professor of Medicine, and in consequence has vacated the Professorship of Pathology and Morbid Anatomy, which will shortly be filled up.

Dr. Watson Williams has been appointed Lecturer on Diseases of the Nose and Throat.

THE Annual Report of the Faculty of Medicine contains a record of many changes, and is of much interest. We therefore give it *in extenso*, as read by Professor E. Fawcett (Dean of the Faculty):—

"The past year, unlike its predecessor, has witnessed many changes in the staff of the Medical Faculty, and overshadowing all in importance is the resignation of one whom we are accustomed to associate with this Report, brought about by an occurrence as sad as it is the lot of any man to experience. For a period of some twenty-six years Dr. Markham Skerritt, our late Dean and Joint Professor of Medicine in this Faculty, has been prominently associated with medical education in this city. He served as secretary to the old Bristol Medical School for some fourteen years, and on the amalgamation of that School with the University College in 1893, Dr. Skerritt became first Dean of the Medical Faculty of that College, in which capacity he acted until his recent resignation. Not only had he acted as Dean during that period, but he had held the Joint Professorship of Medicine since its foundation. His resignation was received with much regret by the Faculty of Medicine, and they have placed on record their high appreciation of the ability and zeal with which Dr. Skerritt has filled these offices, and particularly their recognition of the great services he has rendered to the Bristol Medical School and University College by the continued interest and work he has given to the post of Dean of the Faculty, and, moreover, they have felt and expressed their great sympathy with him in the cause of his resignation. His association with the Faculty will always remain one of those memories which it is pleasant to recall. The Faculty has also to record during the year the resignation of Dr. J. E. Shaw, who for a time was Joint Professor of Medicine; and of Dr. A. B. Prowse, who for seventeen years held the combined Lectureships of Pharmacology and Therapeutics and Materia Medica and Practical Pharmacy with marked success as a lecturer; of Dr. Michell Clarke as Professor of Pathology; and of Dr. J. O. Symes and Mr. Carwardine as Demonstrators of Anatomy. The Faculty very much appreciate the services these gentlemen have rendered to the school, and have been fortunate in retaining the valuable services of Dr. Michell Clarke as Joint Professor of Medicine in succession to Dr. Markham Skerritt. Dr. Edgeworth has succeeded Dr. J. E. Shaw as Joint Professor of Medicine; Dr. Newman Neild and Mr. O. C. M. Davis, B.Sc., have succeeded Dr. Prowse, it having been felt desirable that the Lectureships in Pharmacology and Therapeutics and Materia Medica and Practical Pharmacy should be held by different individuals. Dr. Newman Neild becomes Lecturer on Pharmacology and Therapeutics, whilst Mr. Davis is appointed Lecturer on Materia Medica and Practical Pharmacy. Dr. E. W. Hey Groves and Dr. S. V. Stock were appointed Demonstrators of Anatomy in succession to Dr. Symes and Mr. Carwardine. The vacant Chair of Pathology is as yet unfilled. Changes of another nature have to be recorded. The greatest satisfaction is felt by the Faculty at the great improvements in connection with the medical wing of the University College. The anatomical department has benefitted materially. Not only has the dissecting room been transformed almost beyond recognition, but a new suite of rooms has been added. The bacteriological department is now provided with an independent building which is fast approaching completion. The Faculty is much gratified at the great successes which have been obtained by the students of this College during the past year at the examinations of the various qualifying bodies and by the excellent work which has been done in the school."

**Bristol Medico-Chirurgical Society.**

THE annual meeting of this active and prosperous Society was held on October 11th, 1905, and we have the pleasure to direct especial attention to the

Presidential Address of Mr. John Dacre (page 289). The new version of the old Arabian legend of Zadig and the King's horse should in future be rememberd here as Dacre and the stag. The President's views on the cultivation of the artistic side of the profession should give a new interest to the routine of daily medical life. Of the long series of Presidential Addresses since the year 1874 no one of them has been of more interest or more worthy of serious study.

**Tuberculosis and Immunity.**

THE meeting of the International Congress on Tuberculosis at Paris has again directed especial attention to these subjects, and much has been written on the supposed discovery of a cure announced in the public journals and associated with the name of Professor von Behring, whose work with regard to diphtheria now meets with universal acceptance, and whose expectations with regard to the therapeutic efficacy of his new remedy will be waited for with the greatest anxiety. His book, which should appear next year, will give the evidence obtained by himself and others of the innocuous nature and of the therapeutic value of the new preparation. Meanwhile he wishes that his experiments on animals should be controlled by observers in other laboratories, and that the efficacy and innocuous nature of his new remedy should be established by clinicians. We sincerely hope that the misrepresentations of the lay press, resulting from the sensational method of announcement of his remedy, will not raise such hopes as will lead to disastrous disappointment in the future.

Meanwhile Professor von Behring's method seems to be grounded on the production of a cellular immunity induced by some constituent of the tubercle bacillus. It is difficult to follow the details of the statement made to the Paris Congress, and the literature on immunity has become so increasingly and appallingly complicated that papers on immunity are becoming only intelligible to the few; hence a study of the subject becomes more and more imperative, and the foundation of the superstructure is phagocytosis.

The translation by F. G. Binnie (*Immunity in Infectious Diseases*, by Elie Metchnikoff) of the great work on the subject, just published by the Cambridge University Press, should do much to disseminate a better knowledge of the theory of immunity than as yet prevails. The leading article in the *British Medical Journal* of October 21st, 1905, gives an excellent outline of Metchnikoff's views.

**Proposed National League for Physical Education and Improvement.**

Miss Theodora Johnson's scheme for the establishment of systematic physical education throughout the empire is being gradually worked out. Many meetings have been held, and the importance of the subject is becoming more generally recognised. The objects of the League are stated as follows:—

"It is obvious that as the causes of physical imperfection are so numerous and act upon such numbers of people and over such a wide area, the means to combat these causes must also be various in kind, and must act over the whole length and breadth of the country.

"A great number of agencies are already at work giving instruction in the rules of health, in domestic hygiene and housekeeping, in cooking, and in the care of nursing mothers and in the feeding of children. Others are trying to lessen the consumption of alcohol by supplying nutritious food in its place and in other ways. Others again are trying to improve the physique of the rising generation by supplying gymnasia in town or country; by organising associations for physical exercise, such as cricket, football, drill associations, cadet corps, boys' brigades, rifle clubs, volunteer corps, and girls' clubs; by improving houses and purifying air.

"In the social class immediately above the lowest class of the population there are boys and girls whose occupations are sedentary and their wages small. They cannot afford to join tennis, cricket, football, and similar clubs. For these young and growing persons more playgrounds should exist, gymnasia and swimming baths should be available at a nominal cost. Already many municipalities are converting the swimming baths used only in the summer months into a gymnasia for the winter months.

"The numerous associations which are all working for the common object of increasing the physical development and well-being of our population are at present under the disadvantage of not knowing what the others are doing, and of being even unaware of their very existence."

A scheme of very considerable proportions is being organised, and a long list of influential supporters has already been secured. It is proposed that the subject shall be brought to the notice of the public of the West of England by means of a meeting to be held in the Colston Hall, on February 12th, 1906, the Lord Bishop of Bristol in the Chair.

**Colour and Race.** THE sixth annual Huxley Lecture of the Anthropological Institute was given on October 31st by the ex-President of the Institute, Dr. John Beddoe, F.R.S., who, under the above title, gave an account of the circumstances which determine the colour of a nation's hair and the hue of a people's eyes. He dealt mainly with the problems relating to Central Europe and the British Islands. After adverting to various influences, such as heat, humidity, and different diseases in causing selection of brunettes in certain localities, he explained in detail a series of colour maps which had been prepared from the elaborate statistics compiled by the Belgian, German, and Austrian Statistical Bureaux. The Huxley Memorial Medal was then presented to the venerable anthropologist, much of whose scientific and literary work was done in our midst, and who delivered the Long Fox Lecture in the University College of Bristol in 1904.

